# Biochemical, hematologic, and skeletal features associated with underweight, overweight, and eating disorders in young Korean women: A population-based study

**DOI:** 10.3389/fpsyt.2022.941043

**Published:** 2022-11-25

**Authors:** Zhen An, Kyung-Hee Kim, Mirihae Kim, Youl-Ri Kim

**Affiliations:** ^1^Institute of Eating Disorders and Mental Health, Inje University, Seoul, South Korea; ^2^Department of Food and Nutrition, Duksung Women's University, Seoul, South Korea; ^3^Department of Psychology, College of Social Science, Duksung Women's University, Seoul, South Korea; ^4^Department of Neuropsychiatry, Seoul Paik Hospital, Inje University, Seoul, South Korea

**Keywords:** underweight, anorexia nervosa, bulimia nervosa, overweight, obesity, medical characteristics, extreme weight, abnormal weight

## Abstract

**Background:**

Extreme weight conditions in young women are associated with adverse health outcomes. Closely linked with extreme weight status, eating disorders (EDs) are associated with several medical complications and high mortality rates.

**Objective:**

The study aimed to investigate the biochemical, hematologic, and skeletal features of young Korean women with underweight (UW) and overweight/obesity (OW) conditions, and patients with anorexia nervosa (AN) and bulimia nervosa (BN) compared to women with normal-weight (NW).

**Method:**

A total of 808 women (mean age 22.3 ± 3.4 years) were recruited for the study, including 144 with UW status [body mass index (BMI) < 18.5 kg/m^2^], 364 with NW, 137 with OW or obesity (27 with obesity; BMI ≥ 25 kg/m^2^), 63 patients with anorexia nervosa (AN), and 100 with bulimia nervosa (BN). We measured blood pressure and performed biochemical, hematologic and bone mineral density (BMD) evaluations at the lumbar and femoral neck.

**Results:**

Blood pressure and triiodothyronine levels were found to be lower in both ED groups and higher in the OW group, but no difference in the UW group, compared to the NW group. The aminotransferases and total cholesterol levels were higher in the ED and OW groups, compared to the NW group. Blood cell counts were decreased in the AN group, while increased in the OW group, compared to the NW group. Blood urea nitrogen was elevated in both ED groups. The UW and AN groups had lower BMD, whereas the OW group had higher BMD, compared to the NW group.

**Conclusion:**

Our findings suggested that both ED groups were associated with decreases in the resting energy expenditure. OW status was associated with a risk of metabolic syndrome, and UW status with lower BMD in young women. Overall, the medical parameters in Korean patients with ED were similar to the patterns reported in Western samples in previous studies, with few exceptions such as potassium level in BN.

## Introduction

Abnormal weight conditions and problematic eating behaviors have become prevalent in Asian countries (1). In Korea, the polarization of weight status has been observed in young women in the last two decades (2). While the prevalence of obesity in Korean women in their 20s has increased rapidly since the 1990s (11.6% in 1998 and 22.8% in 2020), the prevalence of underweight (UW) conditions was reported to be over three times higher in young women than in older women (2). Thus, special attention to weight-related health problems in young women in Korea is needed.

Both overweight (OW) and UW conditions in young women of reproductive age are associated with high present and future burdens of disease. In women, adipocytes play an important role in the production and metabolism of estrogen (3), and 17–22% of body weight as adipose tissue is required for the onset of menarche (4). Abnormal body weight leads to dysregulation of the female reproductive hormonal system, which in turn, increases the risk of infertility (5). In addition to its detrimental impact on reproductive health, being UW poses a high risk to skeletal health, leading to low bone mineral density (BMD) in young adult women (6). The adverse health effects of OW status also extend to other systems including abnormalities in cardiovascular and hepatic functions [e.g., (7, 8)].

Eating disorders (EDs) are a common clinical problem among young women in Asian societies (9). Undergraduate Korean women on a diet are up to 15 times more likely to develop an ED than those who are not on a diet (10). The core features of anorexia nervosa (AN), an ED with the highest mortality among psychiatric illnesses (11), are the pursuit of weight loss *via* restrained eating and the resultant low body weight (12). The nutritional compromise of AN as a result of restricted food intake increases medical risk and mortality. Bulimia nervosa (BN), another form of ED, is characterized by episodes of excessive overeating and the subsequent compensatory behaviors (12), and is at risk of developing medical complications (13). The medical risks of EDs affect a broad range of systems including cardiovascular, endocrine, hematopoietic, hepatic, and renal systems due to weight loss and malnutrition in AN, and binge-eating followed by compensatory purging behaviors in BN (14–16). It has been suggested that disordered eating, which manifests itself as EDs, and obesity have commonalities in their etiologies (17), although being overweight or obese *per se* does not indicate an ED. Therefore, investigations into the metabolic differences between EDs and abnormal weight are needed.

Several studies have explored ethnic and cultural differences in ED-related behaviors and ED prevalence, demonstrating similarities and differences between Western and non-Western populations [e.g., (18–22)]. However, there is few research comparing medical status between the populations. Identifying medical similarities and differences in patients with EDs in different ethnic populations would promote the development of effective ethnicity-specific interventions. In addition, the medical status of patients with abnormal weight conditions and EDs has not been extensively compared. Understanding the similarities and differences between the conditions and disorders would help comparing the medical status of people with abnormal weight conditions in Western and non-Western populations in future studies.

The aim of this study was to investigate the biochemical, hematologic, and BMD features in young female college students with UW and OW conditions, and patients with AN and BN, and to examine how they differed from young women with NW. Also, given the impact of ethnicity and culture on the prevalence of ED and symptoms, we were interested in how the clinical findings in our Korean samples would differ from the previous findings in Western samples.

## Methods

### Participants

This was a case-controlled population-based study with a cross-sectional design that aimed to examine the medical conditions of young women with extreme weight and EDs in South Korea. Female university students were recruited from 14 universities in Seoul metropolitan areas *via* research advertisements on their social networking websites from August to December 2016. Patients who had AN or BN were recruited from the ED outpatient clinic in Seoul Paik Hospital during the same period. Parts of the study were previously published (23–25). The groups were defined as underweight (UW, BMI; body mass index < 18 kg/m^2^), normal-weight (NW, 18 kg/m^2^ ≤ BMI < 25 kg/m^2^), and overweight or obese (OW, BMI ≥ 25 kg/m^2^) according to the conventional World Health Organization (WHO) classification (26) instead of the Asia-Pacific regional guidelines (27), and AN and BN based on the Diagnostic and Statistical Manuals of Mental Disorders, Fifth Edition (12). We included participants with obesity (*n* = 27; BMI > 30 kg/m^2^) in the OW group for analysis. Participants had to be female and ≥16 years to be included in the study. The exclusion criteria were (1) severe physical illnesses related to abnormal weight requiring treatment (e.g., diabetes, thyroid problems, and cystic fibrosis); (2) substance use disorder or alcohol dependence, (3) learning difficulties, and (4) pregnancy. University students were included in the study only if they did not report any diagnosis of ED in a self-report. A total of 808 young women enrolled in undergraduate or graduate programs participated in the study. The mean age was 21.96 (SD = 2.58) years in the NW group, 22.41 (SD = 2.72) years in the UW group, and 22.88 (SD = 3.27) years in the OW group. The mean age was 22.57 (SD = 5.47) years in the AN group and 22.37 (SD = 4.74) years in the BN group. Among the students, 364 were classified as NW, 144 as UW, and 137 as OW (110 with OW and 27 with obesity). In the AN group, 26 were extreme, 8 were severe, 11 were moderate, and 17 were mild according to the severity specifier (12). In the BN group, 10 were extreme, 14 were severe, 52 were moderate, and 24 were mild (12).

The participants provided written informed consent after receiving information on the study. Parental consent was obtained for the participants aged under 18 years. This study was approved by the Institutional Review Board (IRB) of Inje University (IRB No. INJE 2016-01-003-002).

### Measures

A demographic and clinical questionnaire assessed the current and highest- and lowest-ever BMI, age, and the age at menarche. BMI [weight in kilogram/(height in meter)^2^] was calculated after measuring the participant's height and weight when wearing light clothes, with shoes and belongings taken off. Subjective health and the frequency of menstruation in 3 months were evaluated by self-reports. Systolic and diastolic blood pressure was measured using an electronic manometre. Fasting blood samples were collected from the participants in a seated position in the morning for laboratory analysis. The blood samples were screened for abnormalities in red blood cell counts, hemoglobin levels, hematocrit levels, white blood cell counts, and platelet counts. Biochemical data including sodium, potassium, aspartate aminotransferase (AST), alanine aminotransferase (ALT), albumin, glucose, blood urea nitrogen (BUN), creatinine, cholesterol, thyroid-stimulating hormone (TSH), and triiodothyronine (T3) levels were collected. BMD was measured by dual X-ray absorptiometry at the lumbar spine (L1–L4) and the right femoral neck. The BMD levels are presented as grams per centimeter squared. T-scores (the number of standard deviations below the mean value of young Korean women) and z-scores (comparison of BMD with a reference Korean population matched for age and sex) were calculated. Laboratory data quality control programs monitored laboratory performance as well as dual energy-ray absorptiometry performed for bone density (DXA, Lunar, GE Healthcare, USA).

### Statistical analysis

The demographic and clinical characteristics of the participants were analyzed by one-way analysis of variance (ANOVA) with a post-hoc Scheffe comparison using the NW group as the reference group. To test for the differences in biochemical and hematologic parameters, and BMD among the groups (UW, OW, AN, and BN groups), we used the logistic regression model with the group as the response variable and the NW group as the reference. Statistical significance was set at the 5% level (*p* < 0.05). All data were analyzed by SPSS 23.0 statistics software (SPSS Inc., New York, NY, USA).

## Results

### Demographic and clinical characteristics

The demographic and clinical characteristics of the participants are presented in Table 1. The UW and AN groups had lower current BMI, whereas it was higher in the OW group. The lowest and highest BMI followed the same pattern except that the BN group had higher BMI than the NW group in the highest BMI, and the AN group's highest BMI did not differ from that of the NW group. There was a difference in the age at menarche among groups, in which the UW group tended to reach menarche at older ages than the NW group. The AN group menstruated less frequently in 3 months than the NW group. Regarding subjective health conditions, the OW, AN, and BN groups reported their conditions to be worse than the NW group.

**Table 1:** Demographic and clinical characteristics of young women with normal-weight, underweight, and overweight status, and patients with eating disorders.

	**NW (*n* = 364)**	**UW (*n* = 144)**	**OW (*n* = 137)**	**AN (*n* = 63)**	**BN (*n* = 100)**	**ANOVA**			**Post hoc Scheffe comparisons with NW**			
						***F***	***df***	***p***	**UW**	**OW**	**AN**	**BN**
									***p***	***p***	***p***	***p***
Age (years), mean (SD)	21.96 (2.58)	22.41 (2.72)	22.88 (3.27)	22.57 (5.47)	22.43 (4.74)	2.12	4, 803	0.076	0.763	0.116	0.776	0.820
BMI kg/m^2^, mean (SD)												
Current	21.01 (1.59)	17.30 (0.78)	28.10 (3.37)	15.57 (2.37)	21.21 (2.93)	573.17	4, 798	<0.001	<0.001	<0.001	<0.001	0.953
Lowest ever	18.91 (1.79)	16.64 (3.86)	22.52 (2.62)	13.54 (2.55)	18.53 (2.84)	136.01	4, 720	<0.001	<0.001	<0.001	<0.001	0.935
Highest ever	22.43 (2.34)	19.56 (4.02)	29.29 (4.08)	21.08 (4.59)	25.68 (5.28)	158.90	4, 719	<0.001	<0.001	<0.001	0.245	<0.001
Age at menarche (years)	12.43 (1.51)	12.90 (1.49)	12.10 (1.84)	12.84 (1.82)	12.84 (1.62)	5.65	4, 727	<0.001	0.061	0.376	0.638	0.585
Menstruation times for 3 months	2.77 (0.55)	2.66 (0.87)	2.60 (0.87)	1.21 (1.47)	2.44 (1.17)	37.45	4, 728	<0.001	0.73	0.32	<0.001	0.12
Subjective health	3.07 (0.74)	3.00 (0.72)	2.60 (0.73)	2.38 (0.98)	2.65 (0.78)	17.26	4, 735	<0.001	0.870	<0.001	<0.001	0.003

Date are shown as mean (SD), otherwise specified. p-value < 0.05 is defined as significant. NW, normal-weight; UW, underweight; OW, overweight; AN, anorexia nervosa; BN, bulimia nervosa; BMI, body mass index.

### Blood pressure and biochemical data

Blood pressure and biochemical parameters of the participants are presented in Table 2. The OW and AN groups showed an opposite pattern in both systolic and diastolic blood pressure. Blood pressure was higher in the OW group and lower in the AN group compared to the NW group. In the BN group, diastolic blood pressure was lower than in the NW group. The UW group had higher levels of AST and albumin, and lower levels of potassium, glucose, and creatinine than the NW group. The OW group displayed higher levels of potassium, AST, ALT, glucose, cholesterol, TSH, and T3 compared to the NW group. The AN group had higher levels of AST, ALT, BUN, and cholesterol, and lower levels of potassium, glucose, and T3. The levels of AST, ALT, BUN, creatinine and cholesterol were higher, and the level of T3 was lower in the BN group compared to the NW group.

**Table 2:** Blood pressure and biochemical data of young women with underweight and overweight status, and patients with eating disorders compared to women with normal-weight.

	**NW (*n* = 364)**	**UW (*n* = 144)**	**OW (*n* = 137)**	**AN (*n* = 63)**	**BN (*n* = 100)**	**NW vs. UW**		**NW vs. OW**		**NW vs. AN**		**NW vs. BN**	
						**Exp (B) (95%CI)**	***P***	**Exp (B) (95%CI)**	***P***	**Exp (B) (95%CI)**	***P***	**Exp (B) (95%CI)**	***P***
**Blood pressure**
Systolic (mmHg)	107.85 (12.76)	105.51 (13.95)	121.40 (14.06)	94.19 (12.60)	103.67 (11.76)	0.99 (0.97–1.00)	0.071	1.07 (1.05–1.09)	<0.001	0.90 (0.86–0.94)	<0.001	0.97 (0.95–1.00)	0.060
Diastolic (mmHg)	69.02 (9.06)	68.60 (7.95)	75.80 (11.13)	61.81 (7.85)	65.78 (9.67)	1.00 (0.97–1.02)	0.645	1.07 (1.05–1.10)	<0.001	0.90 (0.85–0.96)	<0.001	0.96 (0.92–1.00)	0.040
Na (mEq/L)	138.03 (1.43)	138.03 (1.73)	137.77 (1.44)	138.44 (2.96)	137.61 (1.63)	1.00 (0.88–1.13)	0.974	0.91 (0.80–1.02)	0.103	1.17 (0.97–1.43)	0.107	0.86 (0.73–1.02)	0.079
K (mEq/L)	3.94 (0.26)	3.89 (0.29)	3.96 (0.26)	3.84 (0.44)	3.92 (0.29)	0.48 (0.24–0.96)	0.039	1.09 (0.54–2.18)	<0.001	0.25 (0.08–0.74)	0.012	0.71 (0.25–2.04)	0.528
AST (U/L)	18.41 (4.21)	19.94 (6.37)	21.58 (11.09)	34.65 (23.91)	22.08 (12.04)	1.06 (1.03–1·11)	0.001	1.10 (1.06–1.14)	<0.001	1.15 (1.11–1.20)	<0.001	1.11 (1.10–1.15)	<0.001
ALT (U/L)	11.67 (5.40)	12.39 (8.37)	20.84 (22.15)	29.15 (29.22)	15.67 (10.47)	1.03 (1.00–1.07)	0.150	1.12 (1.08–1.15)	<0.001	1.13 (1.09–1.16)	<0.001	1.09 (1.05–1.13)	<0.001
Albumin (g/dL)	4.61 (0.23)	4.67 (0.22)	4.56 (0.21)	4.61 (0.44)	4.60 (0.23)	3.25 (1.39–7.59)	0.006	0.50 (0.23–1.09)	0.081	1.04 (0.29–3.78)	0.95	0.84 (0.25–2.81)	0.774
Glucose (mg/dL)	92.39 (14.42)	89.973 (7.00)	96.66 (9.96)	82.22 (12.68)	90.43 (9.25)	0.96 (0.93–0.98)	0.001	1.04 (1.01–1.07)	0.004	0.86 (0.83–0.90)	<0.001	0.96 (0.93–1.00)	0.070
BUN (mg/dL)	10.74 (2.66)	10.36 (2.68)	10.79 (2.60)	12.04 (4.45)	12.75 (4.06)	0.95 (0.88–1.02)	0.148	1.01 (0.94–1.08)	0.870	1.14 (1.04–1.25)	0.004	1.20 (1.10–1.30)	<0.001
Creatinine (mg/dL)	0.77 (0.08)	0.76 (0.08)	0.76 (0.08)	0.77 (0.13)	0.82 (0.11)	0.09 (0.01–0.93)	0.043	0.11 (0.01–1.09)	0.059	0.52 (0.02–18.49)	0.719	174.31 (7.65–3974.67)	0.001
Cholesterol (mg/dL)	174.51 (27.99)	175.99 (33.47)	181.10 (32.43)	192.57 (46.65)	193.61 (27.42)	1.00 (1.00–1.01)	0.616	1.01 (1.00–1.01)	0.032	1.02 (1.01–1.03)	<0.001	1.02 (1.01–1.03)	<0.001
TSH (μg/mL)	1.64 (0.99)	1.72 (1.06)	2.07 (1.29)	1.32 (0.76)	1.50 (0.65)	1.08 (0.77–1.52)	0.645	1.43 (1.03–1.98)	0.033	0.61 (0.37–1.00)	0.051	0.84 (0.56–1.25)	0.386
T3 (pg/mL)	1.03 (0.13)	1.05 (0.14)	1.14 (0.18)	0.75 (0.22)	0.93 (0.19)	3.12 (0.32–30.18)	0.327	108.29 (8.71–1346.70)	<0.001	8.81E-5 (5.25E-6–0.00)	<0.001	0.02 (0.00–0.18)	0.001

Date are shown as mean (SD). p-value < 0.05 is defined as significant. NW, normal-weight; UW, underweight; OW, overweight; AN, anorexia nervosa; BN, bulimia nervosa; AST, aspartate aminotransferase; ALT, alanine aminotransferase; BUN, blood urea nitrogen; TSH, thyroid stimulating hormone; T3, triiodothyronine.

### Hematologic status

The hematologic parameters of the participants are shown in Table 3. The OW group displayed higher red blood cell, white blood cell, and platelet counts as well as higher hemoglobin and hematocrit levels than the NW group. Conversely, all parameters in the AN group were lower. The UW and BN groups differed from the NW group in white blood cell counts and red blood cell counts, respectively, with lower levels compared to the NW group.

**Table 3:** Hematologic parameters in young women with underweight and overweight status, and patients with eating disorders compared to women with normal-weight.

	**NW (*n*= 364)**	**UW (*n* = 144)**	**OW (*n* = 137)**	**AN (*n* = 63)**	**BN (*n* = 100)**	**NW vs. UW**		**NW vs. OW**		**NW vs. AN**		**NW vs. BN**	
						**Exp (B) (95%CI)**	***P***	**Exp (B) (95%CI)**	***P***	**Exp (B) (95%CI)**	***P***	**Exp (B) (95%CI)**	***P***
RBC (10^6^/mm^3^)	4.42 (0.29)	4.40 (0.34)	4.53 (0.30)	4.06 (0.51)	4.30 (0.29)	0.83 (0.44–1.56)	0.566	3.46 (1.77–6.76)	<0.001	0.06 (0.02–0.15)	<0.001	0.32 (0.13–0.79)	0.013
Hb (g/DI)	13.01 (0.98)	13.02 (0.93)	13.25 (0.92)	12.45 (1.50)	12.94 (1.02)	1.01 (0.83–1.23)	0.925	1.31 (1.06–1.62)	0.014	0.65 (0.50–0.84)	0.001	0.94 (0.70–1.25)	0.646
Hct (%)	39.18 (2.49)	39.26 (2.53)	39.85 (2.48)	37.29 (4.22)	38.62 (2.46)	1.01 (0.94–1·09)	0.77	1.11 (1.03–1.21)	0.009	0.80 (0.73–0.89)	<0.001	0.92 (0.83–1.03)	0.148
WBC (10^3^/mm^3^)	5.98 (1.53)	5.40 (1.37)	6.65 (1.91)	4.59 (1.49)	5.99 (1.68)	0.76 (0.66–0.88)	<0.001	1.25 (1.11–1.39)	<0.001	0.45 (0.34–0.59)	<0.001	1.00 (0.84–1.21)	0.964
Platelet (10^3^/mm^3^)	264.23 (51.17)	251.74 (51.95)	289.88 (62.12)	226.39 (70.66)	262.65 (58.11)	1.00 (1.00–1.00)	0.019	1.01 (1.00–1·01)	<0.001	0.99 (0.98–0.99)	<0.001	1.00 (0.99–1.01)	0.845

Date are shown as mean (SD). p-value < 0.05 is defined as significant. NW, normal-weight; UW, underweight; OW, overweight; AN, anorexia nervosa; BN, bulimia nervosa; RBC, red blood cell count; Hb, hemoglobin; Hct, hematocrit; WBC, white blood cell count.

### Skeletal health

Table 4 demonstrates the BMD of the participants at the measured sites. The UW group had lower density at the lumbar spine and the right femoral neck with lower t-scores, whereas the OW group had higher density at both sites with higher t-scores compared to the NW group. All BMD measures were lower in the AN group than in the NW group. There was no difference in the BMD between the BN group and the NW group.

**Table 4:** Bone mineral density at lumbar spine and femoral neck in young women with underweight and overweight status, and patients with eating disorders compared to women with normal-weight.

	**NW (*n* = 364)**	**UW (*n* = 144)**	**OW (*n* = 137)**	**AN (*n* = 63)**	**BN (*n* = 100)**	**NW vs U W**		**NW vs. OW**		**NW vs. AN**		**NW vs. BN**	
						**Exp (B) (95%CI)**	***P***	**Exp (B) (95%CI)**	***P***	**Exp (B) (95%CI)**	***P***	**Exp (B) (95%CI)**	***P***
L1–L4 (g/cm^2^)	1.16 (0.12)	1.09 (0.12)	1.25 (0.12)	0.99 (0.23)	1.16 (0.15)	0.03 (0.00–0.33)	0.005	84.74 (5.08–1413.00)	0.002	0.00 (6.30E-6–0.00)	<0.001	1.26 (0.09–18.62)	0.87
L1–L4 (T score)	0.09 (0.97)	−0.47 (1.02)	0.86 (0.99)	−1.53 (1.44)	0.11 (1.21)	0.62 (0.45–0.86)	0.004	2.00 (1.34–2.87)	<0.001	0.26 (0.17–0.40)	<0.001	1.02 (0.73–1.44)	0.906
Rt FeN (g/cm^2^)	0.95 (0.14)	0.91 (0.10)	1.05 (0.12)	0.80 (0.13)	0.97 (0.13)	0.05 (0.00–1.09)	0.057	829.47 (31.07–22147.81)	<0.001	3.14E-5 (6.47E-7–0.00)	<0.001	4.55 (0.20–101.43)	0.338
Rt FeN (T score)	0.14 (0.92)	−0.28 (0.82)	0.90 (1.04)	−1.22 (1.05)	0.22 (1.12)	0.62 (0.42–0.90)	0.012	2.09 (1.41–3.09)	<0.001	0.19 (0.11–0.33)	<0.001	1.10 (0.75–1.60)	0.638

Date are shown as mean (SD). p-value < 0.05 is defined as significant. NW, normal-weight; UW, underweight; OW, overweight; AN, anorexia nervosa; BN, bulimia nervosa; Rt FeN, right femoral neck.

## Discussion

The incidences of eating disorders and extreme weight conditions are increasing in Korean young women. We previously reported factors associated with extreme weight status and eating disorders in young Korean women, regarding nutrients consumption and eating behaviors (25). Here, we evaluated the medical features associated with extreme weight status and eating disorders in young women. Elevated AST, ALT, and cholesterol levels were observed in both ED and the OW groups compared to the NW group. Both ED groups showed reduced T3 levels and higher serum urea nitrogen compared to the NW group. The hematologic parameters were lower in the AN group, and BMD was lower in the UW and AN groups, compared to the NW group. Whereas most medical parameters remained within the reference range in the young women with UW, the OW and ED groups reported their conditions to be worse than those reported by the NW group.

Blood pressure was found to be lower in both AN and BN groups, and higher in the OW group, but no difference in the UW group, compared to the NW group. The findings suggested changes in the hemodynamic status may be attributable to reduced energy intake than weight status. The energy intake and expenditure among the groups were also reflected in the T3 level with similar direction to those in blood pressure. We previously reported T3 as a factor distinguishing constitutional thinness from AN (28). Free T3 has been reported to constitute a strong significant tool distinguishing constitutional thinness from AN (29, 30). As elevated thyroid hormone concentrations increase the resting energy expenditure, T3 values in or slightly above the upper normal range in OW women seem rather a consequence than a cause of OW since weight loss leads to a normalization of elevated thyroid hormone levels (29).

Abnormalities of liver enzymes and total cholesterol are well recognized in AN and OW/obesity. In the present study, AST, ALT and total cholesterol levels were higher in the AN, BN, and OW groups, compared to the NW group. The elevated levels of AST/ALT in AN are likely attributable to apoptosis (programed liver cell death), as a direct result of malnutrition (31, 32) whereas the findings in the OW group are likely attributable to rapid and excessive deposition of fat in the liver (33), and both mechanisms may explain higher ALT and AST levels in BN patients. Though an elevated level of total cholesterol is a common finding in patients with EDs (34–36) and obesity, the etiology of hypercholesterolemia may result from different mechanism among groups. Hypercholesterolaemia in patients with EDs might result from increased resorption of endogenous cholesterol in patients with AN (37, 38) or from the metabolic alterations followed by cyclical patterns of binging in patients with BN (39). More conclusive evidence that supports AN as an atherogenic condition is needed (40).

Hematologic parameters were reduced in the AN group compared to the NW group, as reported in patients with AN in both Korean (34) and Western (41, 42) populations, with malnutrition and bone marrow hypoplasia as the potential mechanisms.

BMDs were significantly lower in the UW and AN groups compared to women with NW as previously studied in Korean (28, 34, 43) and American women (44, 45). These findings, combined with a systematic review that demonstrated weight gain and subsequent improvements in BMD in patients with AN (46), indicated a positive association between BMI and BMD. Consistent with these findings, young women with OW conditions had higher BMDs than those with NW in our study. This was further supported by meta-analyses that identified OW/obesity as a protective factor for osteoporosis and fractures (47, 48). However, heterogeneity, including ethnicity and the skeletal sites measured, need to be considered (47, 49), along with scarce research on the relationship between obesity and bone health in premenopausal women (50).

Our findings suggest general similarities but some differences from the reports in Western populations. The serum urea nitrogen levels were increased in the AN and BN groups, compared to the NW group, as previously described in Korean (34, 35). On the other hand, there was a report of low level of blood urea in Western populations with AN (51) due to decreased protein uptake and loss of muscle mass. Our findings indicate significant dehydration and the possibility of a risk of pre-renal azotemia in patients with EDs.

Hypokalaemia is the most common electrolytes abnormality found in AN (52). We found decreased potassium in both UW and AN groups, but not in the BN group, compared to the NW group, which was consistent with the previous finding in Korean populations (35). The findings in the Korean patients with BN were different from those in Western populations, in which hypokalaemia is addressed as one of the most common electrolyte abnormalities of BN (13, 16). The difference might be attributed to binging of potassium rich Korean staple foods such as Kim-Chi in Korean patients with BN, the high proportion of outpatients (53) or somewhat corrected nutrition *via* nutritional counseling when the patients participated in the present study.

Strengths of the present study included the recruitment of appropriate samples of young women with extreme weight status based on the global criteria of BMI instead of regional criteria, and the comprehensive assessment of the medical parameters. However, a few limitations need to be addressed when interpreting the results. One was that since the data were drawn from young Korean women, the results cannot be generalized to more diverse groups regarding ethnicity and age. Other limitation was that many patients' nutrient intake and eating behaviors might be somewhat corrected as most of them were undergoing treatment. Lastly, we relied on a self-report rather than an interview to exclude diagnoses of ED from the student sample.

In conclusion, our data on young Korean women with UW, NW, and OW, and patients with AN or BN suggest that both ED groups and the OW group had medical complications, which were expressed biochemically, hematologically, and skeletally. Overall, similar patterns in clinical parameters were observed in the present study in Korean patients with EDs compared to the previous studies in Western populations. However, differences such as the levels of serum urea nitrogen and potassium in patients with AN and BN existed, which might require further research in cultural aspects. This study provides meaningful insights into the comparison of medical parameters between Western and Korean populations with EDs, and between EDs and extreme weight conditions.

## Data availability statement

The raw data supporting the conclusions of this article will be made available by the authors, without undue reservation.

## Ethics statement

The studies involving human participants were reviewed and approved by Institutional Review Board of Inje University. Written informed consent to participate in this study was provided by the participants' legal guardian/next of kin.

## Author contributions

Y-RK designed, conceptualized the study, and critically revised the manuscript. Y-RK, K-HK, and MK collected the data. ZA and Y-RK analyzed the data and formulated the manuscript. All authors contributed to the manuscript and approved the submitted version.

## Funding

This work was supported by the Korea Centers for Disease Control and Prevention Research Fund [Grant Number: 2016-ER6310-00, 2016] and the National Research Foundation (NRF) of Korea [Grant Number: 2021R1A2C2009668, 2021].

## Conflict of interest

The authors declare that the research was conducted in the absence of any commercial or financial relationships that could be construed as a potential conflict of interest.

## Publisher's note

All claims expressed in this article are solely those of the authors and do not necessarily represent those of their affiliated organizations, or those of the publisher, the editors and the reviewers. Any product that may be evaluated in this article, or claim that may be made by its manufacturer, is not guaranteed or endorsed by the publisher.
